# Investigating the role of the mPGES-PGE₂-EP4 pathway in *Escherichia coli*-induced mastitis in dairy cows: insights for non-antibiotic therapeutic strategies

**DOI:** 10.3389/fvets.2025.1628028

**Published:** 2025-07-01

**Authors:** Xiaolin Yang, Xueqiang Li, Lili Guo, Pengfei Gong, Yinghong Qian, Shuangyi Zhang, Bo Liu, Wenrui Guo, Haixia Bao, Wei Mao

**Affiliations:** ^1^Key Laboratory of Clinical Diagnosis and Treatment Techniques for Animal Disease, Ministry of Agriculture, Inner Mongolia Agricultural University, Hohhot, China; ^2^Laboratory of Veterinary Clinical Pharmacology, College of Veterinary Medicine, Inner Mongolia Agricultural University, Hohhot, China; ^3^Inner Mongolia Bayannaoer City Municipal Center for Disease Control and Prevention, Bayannur, China; ^4^Inner Mongolia Academy of Agricultural & Animal Husbandry Sciences, Hohhot, China

**Keywords:** *Escherichia coli*, mastitis, alternative treatments to antibiotics, mPGES-1 inhibitors, EP4 receptor inhibitor

## Abstract

*Escherichia coli* (*E. coli*) is the primary causative agent of bovine mastitis. Currently, antibiotic therapy remains the cornerstone of mastitis treatment; necessitating the identification of alternative therapeutic options. This study employed *in vitro* cultured bovine bone marrow-derived macrophages (BMDMs) to systematically assess the potential of microsomal prostaglandin e synthase-1 (mPGES-1) inhibitors (MF63, MK886) and EP4 receptor inhibitor (Grapiprant) in modulating inflammatory responses and reducing tissue damage. Cells were pre-treated with mPGES-1 inhibitors and an EP4 receptor inhibitor before infection with *E. coli*. Following infection, extracellular bacteria were removed, and assays—including ELISA, Western blot, and qRT-PCR—were conducted to analyze inflammatory mediators, protein expression, and gene expression. *E. coli* infection significantly induced PGE₂ synthesis in BMDMs, which exacerbated the inflammatory response and tissue damage via NF-κB and MAPK signaling pathways, elevating TNF-*α*, IL-1β, IL-6, and IL-8. Treatment with MF63, MK886 and Grapiprant effectively reduced PGE₂ levels, inhibited NF-κB and MAPK signaling pathways, decreased inflammatory mediators, and enhanced macrophage bactericidal activity, thereby demonstrating potent anti-inflammatory and immunomodulatory effects. Moreover, inhibition of the mPGES-PGE₂-EP4 signaling pathway was found to reduce the expression of damage-associated molecular patterns (HMGB-1 and HABP-2), suggesting alleviation of *E. coli*-induced tissue damage. Based on the role of PGE₂ in mediating immune and inflammatory responses via the EP4 receptor, inhibiting the mPGES-1-PGE₂-EP4 signaling axis to reduce inflammation and tissue damage will facilitate further investigation into the regulatory mechanisms of the PGE₂ signaling axis in the pathogenesis of mastitis. This approach provides a theoretical foundation and experimental basis for the development of alternative anti-inflammatory therapies to replace antibiotics.

## 1 Introduction

Milk is recognized as one of the most significant animal-derived foods worldwide, characterized by its rich composition of high-quality proteins, fats, vitamins, and minerals, and its irreplaceable role in human nutrition (1). The escalating demand for dairy products has prompted modern dairy farming to prioritize increasing milk yield and improving milk quality (2). However, mastitis, a major disease affecting udder health, significantly reduces milk yield, compromises milk quality, and results in substantial economic losses for the dairy industry (3). Given the complex pathogenesis of mastitis, current treatment strategies predominantly rely on antibiotics (4). Yet, prolonged antibiotic use may lead to bacterial resistance and drug residues, posing a threat to food safety (5, 6). Therefore, understanding the pathogenesis of mastitis and exploring safer, more effective alternative therapies are essential for the health of dairy cows and the sustainable development of the dairy industry.

*Escherichia coli* (*E. coli*) is a major pathogen responsible for mastitis among dairy cows (7), utilizing various virulence factors to inflict damage on host cells, induce inflammation, and evade the immune system. These effects compromise the integrity of mammary tissue and exacerbate mastitis progression (8–10). Mastitis does not only reduce milk quality but also facilitates the entry of pathogens and their virulence factors into milk, thereby affecting food safety (11). Therefore, understanding the pathogenic mechanisms of *E. coli*, along with its interaction with the host immune responses, is crucial for preventing mastitis and ensuring dairy product safety.

Macrophages residing in mammary tissue, primarily originating from the bone marrow, are key immune cells in pathogen defense (12). The M1 macrophage phenotype is associated with inflammatory and antitumor functions. Monocytes and macrophages in the bone marrow and peripheral blood are primarily derived from hematopoietic stem cells in the bone marrow; therefore, we used bovine bone marrow-derived macrophages (BMDMs) in the present study (13). Their roles encompass pathogen recognition, phagocytosis, and elimination, while also maintaining tissue homeostasis by regulating inflammatory responses (14, 15). However, under pathological conditions, damage to mammary tissue may result in the disruption of the blood-milk barrier, leading to the excessive release of pro-inflammatory cytokines and exacerbating tissue damage, thereby creating a vicious cycle of inflammatory imbalance (16).

Mastitis is an inflammatory process of the mammary gland that leads to the production of prostaglandins, resulting in increased body and mammary surface temperature (17). Studies have shown that prostaglandins, particularly prostaglandin E_2_ (PGE₂), play a significant role in the pathophysiology and severity of *E. coli*-induced mastitis, especially during the early lactation phase in dairy cows (18, 19). PGE_2_, synthesized during arachidonic acid (AA) metabolism by cyclooxygenase (COX-1/COX-2) and prostaglandin E synthase (PGES), is a key bioactive lipid molecule involved in regulating inflammation, immune responses, and tissue repair (20). It exerts its biological effects through four G protein-coupled receptors (EP1–EP4) (21), with EP4 being particularly critical in immune regulation and tissue damage repair during mastitis (22).

In the pathological process of bovine mastitis, *E. coli* infection of mammary tissue activates the TLR4/NF-κB signaling pathway (23), which subsequently leads to the upregulation of COX-2 expression, promoting the synthesis of PGE₂ and activating the EP4 receptor (24, 25). The PGE₂-EP4 signaling pathway not only influences inflammation regulation and tissue repair, but may also affect the efficacy of antibiotic treatment for inflammation and the development of resistance (26). The study suggests that PGE₂ can upregulate the expression of drug resistance-associated genes through the EP4 receptor, thereby reducing the therapeutic efficacy of antibiotics (27). Therefore, targeting the PGE₂-EP4 signaling pathway may represent a novel strategy for the treatment of mastitis, helping to alleviate inflammatory damage while potentially replacing the use of antibiotics and reducing the risk of resistance.

This study utilized mPGES-1 inhibitors (MF63, MK886) and EP4 receptor inhibitor (Grapiprant) to block the PGE₂-EP4 signaling pathway. MF63—compared to earlier mPGES-1 inhibitors—demonstrates stronger potency and higher selectivity in cellular assays, making it a more promising candidate for targeting PGE₂ inhibition (28). Preclinical studies have shown that MF63 effectively inhibits the synthesis of inflammatory PGE₂ while preserving other prostaglandin biosynthetic pathways, thus minimizing off-target effects and enhancing its therapeutic potential in inflammatory conditions (29). MK-886 also serves as an effective mPGES-1 inhibitor thatobstructs the intracellular biosynthesis of PGE₂ (30). Widely used in experimental models of inflammation, allergy, cancer, and cardiovascular diseases, MK-886 represents an established tool for investigating PGE₂-related pathways (31). Grapiprant, a highly selective EP4 receptor antagonist, inhibits the PGE₂-EP4 signaling pathway and downstream inflammatory responses (32). Unlike traditional nonsteroidal anti-inflammatory drugs (NSAID) that broadly inhibit COX activity, Grapiprant specifically targets the EP4 receptor. Approved for the treatment of osteoarthritis-related pain and inflammation in dogs, Grapiprant has demonstrated efficacy and safety in preclinical and clinical studies, alleviating arthritis inflammation, relieving pain, and improving mobility by blocking EP4-mediated pro-inflammatory signaling (33). Accordingly, it can be speculated that the PGE₂-EP4 signaling pathway may play a crucial role in the treatment of mastitis in dairy cows.

Although there is an abundance of literature on mastitis in dairy cows, data regarding its underlying mechanisms remain insufficient. Given the detrimental effects associated with antibiotic use for the treatment of bovine mastitis in humans, the potential of PGE₂-EP4 inhibitors for managing mastitis has garnered renewed interest. However, the role of PGE₂-EP4 inhibitors in treating mastitis in dairy cows, particularly concerning the underlying molecular mechanisms, has largely been overlooked.

This study aims to investigate the anti-inflammatory effects of PGE₂-EP4 inhibitors in an *E. coli*-induced macrophage model, their impact on phagocytic killing ability against *E. coli*, and the underlying molecular mechanisms.

## 2 Materials and methods

### 2.1 Ethical statement

Bovine ribs were obtained postmortem from adult dairy cows that were slaughtered for commercial food production at Beiya Halal Slaughterhouse (Hohhot, Inner Mongolia, China). No animals were euthanized for the purposes of this study, and no live animal interventions were conducted. As a result, ethical approval was not necessary.

### 2.2 Bacterial strains

A 1 mL suspension of *E. coli* O157 (34, 35) strain (at a concentration of 1 × 10^7^ CFU) preserved in the laboratory was inoculated into 100 mL of Luria-Bertani (LB) broth (Oxoid, Basingstoke, LTD, UK). Incubate the culture at 37°C with shaking at 200 rpm for 12 h, or until the OD_600_ of the culture reaches 0.9. The bacterial suspension was serially diluted and plated onto LB agar. After incubation at 37°C for 18 h, colonies were counted, and the concentration was quantified as CFU/mL.

### 2.3 Culture of bovine BMDMs

The bovine rib marrow cavity was washed with phosphate-buffered saline (PBS), and the cell suspension was filtered through a cell strainer into a 50 mL centrifuge tube (Hyclone, UT, United States). The suspension was centrifuged at 2900 g for 8 min, and the supernatant was discarded. Red blood cells were lysed using red blood cell lysis buffer for 5 min, followed by centrifugation at 1300 g for 8 min. The bone marrow cells were then collected and cultured in Roswell Park Memorial Institute (RPMI) 1,640 medium with 20% fetal bovine serum (Hyclone, UT, United States) and 20 ng/mL macrophage colony-stimulating factor (Kingfisher, MN, United States) at 37°C in a 5% CO_2_ incubator. After 7 days of induction, non-adherent cells were removed, and adherent cells were treated with 1 μg/mL lipopolysaccharide (LPS, PeproTech, NJ, United States) for 24 h to differentiate M0 macrophages into M1 macrophages for subsequent experiments.

### 2.4 Experimental infection and treatment *in vitro*

Cells were treated with mPGES-1 inhibitors (MF63, MK886; Cayman Chemical Company, Ann Arbor, MI, United States) for 24 h and EP4 receptor inhibitor (Grapiprant; MedChemExpress, Shanghai, China) for 4 h, followed by *E. coli* infection. At 1-h post-infection, cells were washed with fresh medium containing 100 μg/mL tobramycin to remove extracellular bacteria (36).

### 2.5 Enzyme-linked immunosorbent assay

After pretreatment with mPGES-1 inhibitors (MF63, MK886) and EP4 receptor inhibitor (Grapiprant), cell supernatants were collected 6 h after *E. coli* infection. The secretion of PGE_2_, TNF-*α*, IL-1β, IL-6, IL-8, and IL-10 in the BMDM supernatant was measured using ELISA kits for bovine PGE_2_ (Cayman Chemical Company, MI, United States), TNF-α, IL-6 (R&D Systems, MN, United States), and IL-1β, IL-8, and IL-10 (Kingfisher Biotech, MN, United States).

### 2.6 Cell viability assay

The MTT assay was used to assess the impact of the drugs on cell viability. BMDM (10⁴ cells per well) were seeded into a 96-well plate and cultured in 180 μL of medium under conditions of 37°C and 5% CO₂. Following treatment with MF63, MK886, and Grapiprant, the MTT assay was performed according to the manufacturer’s instructions (Solarbio, Beijing, China).

### 2.7 Western blot analysis

After pretreatment with mPGES-1 inhibitors (MF63, MK886) and EP4 receptor inhibitor (Grapiprant), cells were collected after 15, 30 and 60 min of *E. coli* infection. Total protein was extracted from treated cells using M-PER Mammalian Protein Extraction Reagent (Thermo Scientific, MA, United States) and quantified with the BCA Protein Assay Kit (Thermo Scientific, IL, United States). An aliquot of 10 μg of total protein was loaded onto each lane, and the samples were separated by 12% SDS-PAGE. Following membrane transfer, the membrane was blocked at room temperature for 1 h and then incubated overnight with primary antibodies at 4°C. The primary antibodies employed included anti-phospho-ERK, anti-ERK, anti-phospho-p38, anti-p38, anti-phospho-NF-κB p65, anti-NF-κB p65 (1:1000, Cell Signaling Technology, MA, United States), and anti-GAPDH (1:1000). The protein bands were visualized using enzyme-linked secondary antibodies and Pierce SuperSignal West Femto Chemiluminescent Substrate (Thermo Scientific, IL, United States). Image analysis was conducted using ImageJ software (version 1.48; NIH, MD, United States).

### 2.8 Quantitative real-time polymerase chain reaction

After pretreatment with mPGES-1 inhibitors (MF63, MK886) and EP4 receptor inhibitor (Grapiprant), cells were collected at 2, 4, and 6 h post-*E. coli* infection. Total RNA was extracted using an RNA extraction kit (Axygen Scientific, CA, United States), and reverse transcription was performed with the RevertAid First Strand cDNA Synthesis Kit (Vazyme, Nanjing, China). Quantitative real-time PCR was conducted on an ABI PCR System (Bio-Rad, Hercules, CA, United States) using FastStart SYBR Green Master (Roche Applied Science, Mannheim, Germany). PCR conditions included 50°C for 2 min, 95°C for 10 min, followed by 40 cycles of 95°C for 15 s and 60°C for 60 s. Primers for quantitative PCR are listed in Table 1. Relative gene expression was normalized to *β*-actin and calculated using the 2 ^−ΔΔCt^ method.

**Table 1:** Primers used in this study.

Accession No.	Gene name	Primer sequence	Amplicon size
NM_173979.3	β-actin	Forward:5'-ATCGGCAATGAGCGGTTC-3' Reverse:5'-CCGTGTTGGCGTAGAGGT-3'	144bp
XM_024551043.1	HMGB-1	Forward:5'-AAGTTCAAGGATCCCAATGCAC-3' Reverse:5'-GCTTATCATCCGCAGCAGTGT-3'	162bp
XM_027528630.1	HABP-2	Forward:5'-TCTGACAACCCTGACTGGTACTAC-3' Reverse:5'-GTGGTAAGGAGGACTCTGAGTAATG-3'	212bp

HMGB-1, High mobility group box-1; HABP-2, Hyaluronic acid binding protein-2.

### 2.9 Microscopy assay of bacterial phagocytosis and tetrazolium dye reduction for bacterial killing

To investigate the effects of mPGES-1 and EP4 treatments on phagocytosis and killing of *E. coli* by BMDM, cells were cultured at a density of 2 × 10^5^ in 35 mm dishes and treated with or without MF63, MK886, and Grapiprant. The cells were co-incubated with 8 μM 1,1′-octadecyl-3,3,3′,3′-tetramethylindodicarbocyanine perchlorate (Thermo Scientific, CA, United States) for 20 min to label cell membranes, and *E. coli* along with Hoechst 33258 for 20 min to label the bacteria. Following this, BMDM were infected with *E. coli* for 0.5 or 2.5 h, fixed with 4% paraformaldehyde, and observed at 400 × magnification using a confocal microscope (LSM 800; Carl Zeiss, Oberkochen, Germany).

### 2.10 Data analysis

Data were analyzed using GraphPad Prism 10 software (GraphPad InStat Software, CA, United States) and expressed as mean ± standard deviation (SD). Statistical significance was determined by one-way or two-way ANOVA with appropriate *post hoc* tests (Tukey’s or Bonferroni). *p* values < 0.05 were considered significant (*p < 0.05; **p < 0.01; ***p < 0.001; ****p < 0.0001).

## 3 Results

### 3.1 Selection of drug concentrations of mPGES-1 inhibitors in *Escherichia coli* infected BMDM

**Figure 1:**
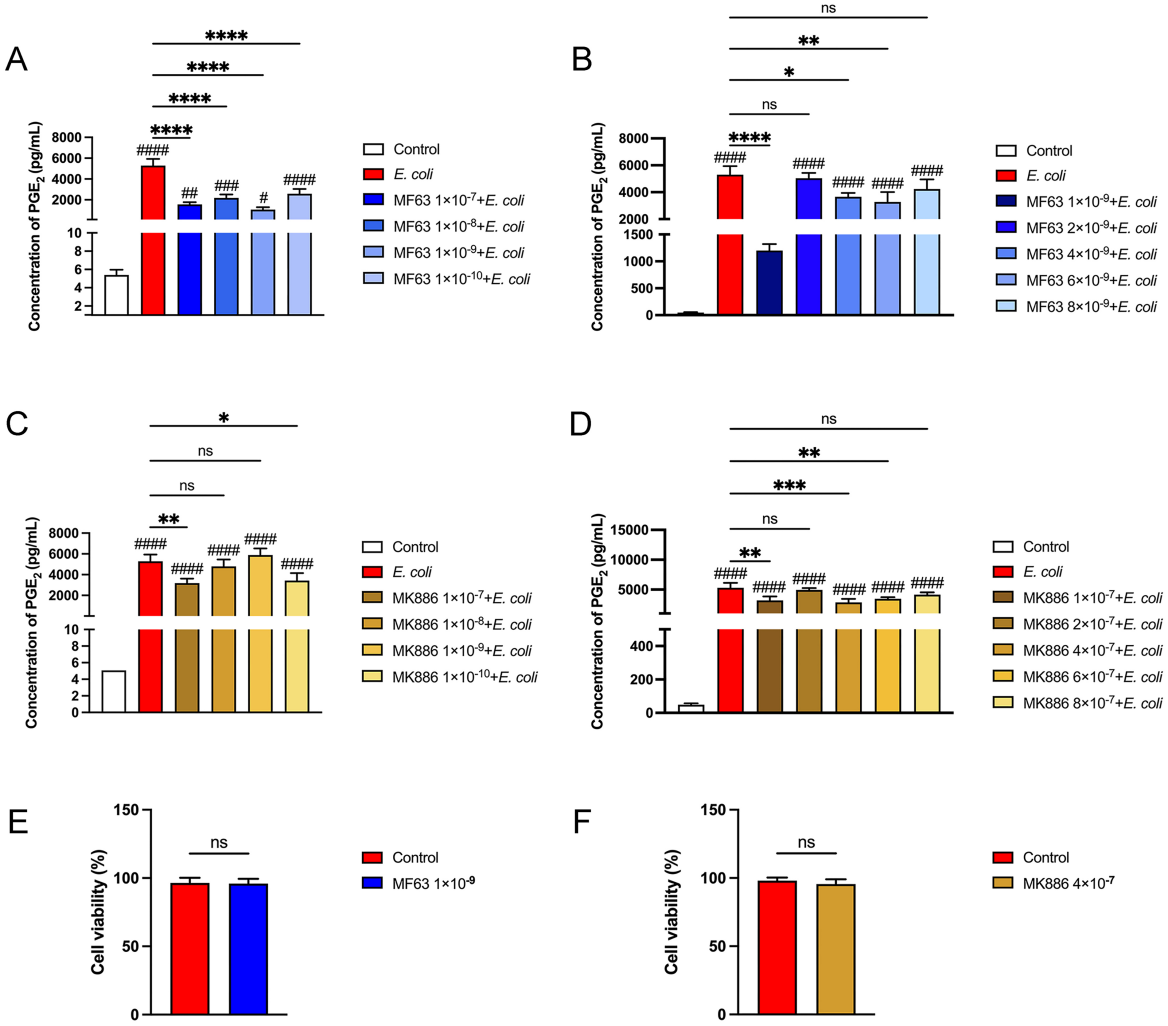
Results of drug selection. **(A,B)** Effect of varying concentrations of MF63 on PGE₂ secretion in *E. coli*-infected BMDMs. **(C,D)** Effect of varying concentrations of MF63 on PGE₂ secretion in *E. coli*-infected BMDMs. **(E)** Cell viability of MF63 (1 × 10^−9^ M)-treated BMDMs was assessed using the MTT assay. **(F)** Cell viability of MK886 (4 × 10^−7^ M)-treated BMDMs was assessed using the MTT assay. Data are expressed as mean ± SD. * *p* < 0.05, ** *p* < 0.01, *** *p* < 0.001, **** *p* < 0.0001 vs. *E. coli* group; ^#^ *p* < 0.05, ^##^ *p* < 0.01 ^###^ *p* < 0.001, ^####^ *p* < 0.0001 vs. control group.

We conducted a series of concentration-gradient experiments to select the optimal drug concentration. The results showed that during *E. coli* infection of BMDM, PGE_2_ secretion was significantly reduced at an MF63 concentration of 1 × 10^−9^ M and an MK886 concentration of 1 × 10^−7^ M within the drug concentration range of 1 × 10^−7^ M to 1 × 10^−10^ M, indicating optimal therapeutic efficacy (Figures 1A,B, *P* < 0.05). Further refinement revealed the lowest secretion of PGE_2_ at a concentrations of 1 × 10^−9^ M and 4 × 10^−7^. Accordingly, MF63 (1 × 10^−9^ M) and MK886 (4 × 10^−7^ M) were chosen as the optimal drug concentration for use in *E. coli*-infected BMDM in subsequent experiments (Figures 1C,D, *P* < 0.05). Cell viability, measured using the MTT assay according to the manufacturer’s instructions, demonstrated no difference between the drug groups (MF63, MK886) and the control group, suggesting a lack of toxic effects on BMDM (Figures 1E,F).

### 3.2 Analysis of cytokines and signaling pathways of *Escherichia coli* infected BMDM by inhibiting the mPGES-1-PGE_2_ axis

**Figure 2:**
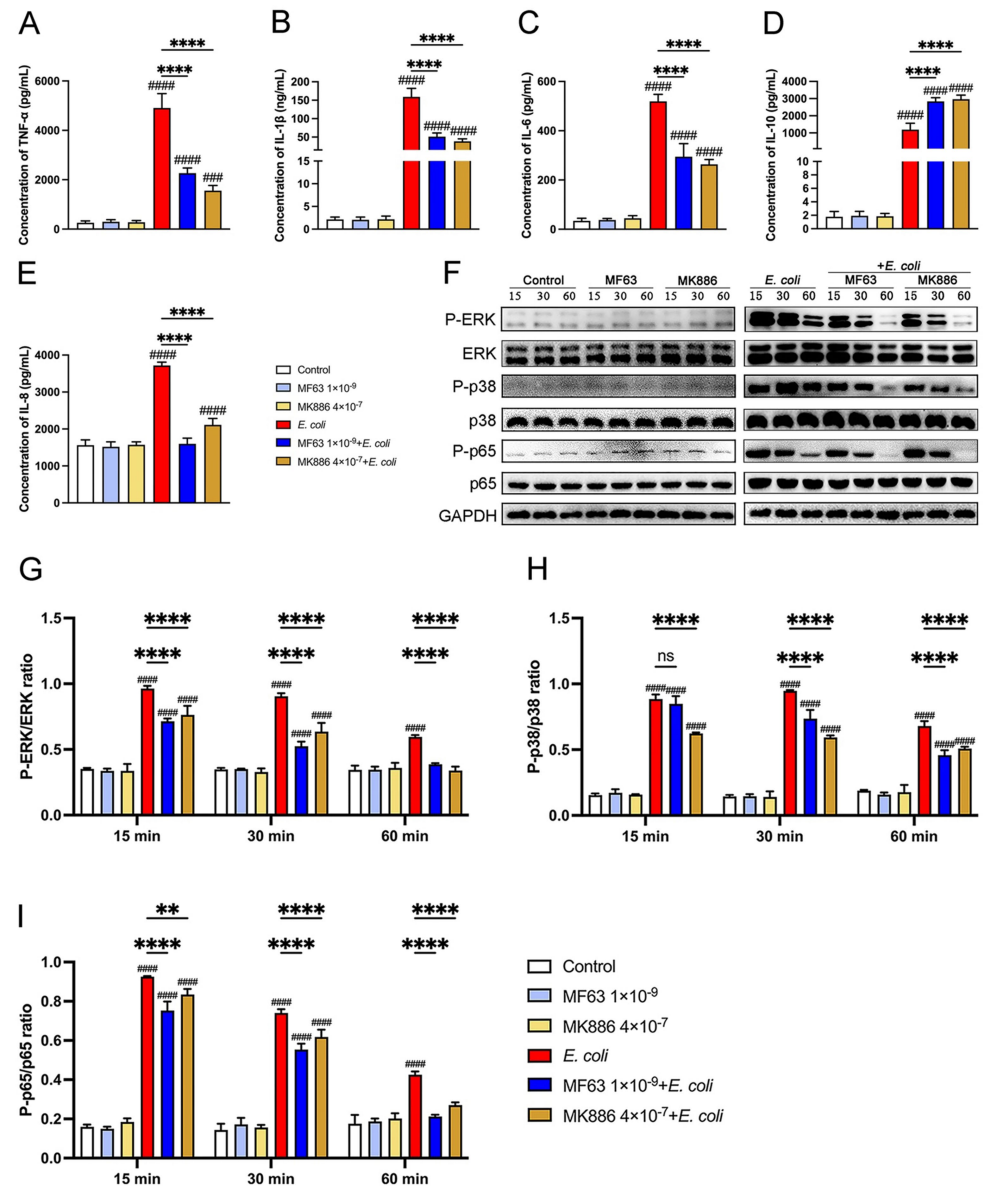
Impact of mPGES-1 inhibitors (MF63 and MK886) on cytokine secretion and signaling pathways in *E. coli*-infected BMDMs. **(A–E)** BMDMs were pretreated with MF63 and MK886 and subsequently infected with *E. coli* at a MOI of 5:1. The secretion levels of TNF-*α*, IL-1*β*, IL-6, IL-10, and IL-8 in the BMDMs culture supernatants were measured by ELISA. **(F–I)** Phosphorylation levels of ERK, p38, and p65 were assessed by western blotting at 15-, 30-, and 60-min post-infection, with GAPDH used as the loading control. Grayscale values were quantified using ImageJ software. Results are presented as mean ± SD from three independent experiments, analyzed by Tukey’s multiple comparisons and two-way ANOVA. Significance is denoted as *p < 0.05, **p < 0.01, ***p < 0.001, ****p < 0.0001 vs *E. coli* group; ^#^*p* < 0.05, ^##^*p* < 0.01 ^###^*p* < 0.001, ^####^*p* < 0.0001 vs Control group.

Cytokines and chemokines function as key mediators in the inflammatory response to bacterial infections. We investigated the effects of mPGES-1 inhibitors (MF63 and MK886) on the production of pro-inflammatory cytokines (TNF-*α*, IL-1β, and IL-6), the anti-inflammatory cytokine IL-10, and the chemokine IL-8 in macrophages infected with *E. coli* at a multiplicity of infection (MOI) of 5:1. As shown in Figures 2A–E, *E. coli* infection significantly increased cytokine and chemokine secretion in BMDM (*p* < 0.0001). However, treatment with MF63 and MK886 markedly reduced the secretion of TNF-α, IL-1β, IL-6, and IL-8, while elevating IL-10 secretion compared to the *E. coli*-infected group (*p* < 0.0001). To further elucidate the underlying mechanism, we examined the activation of the MAPK (ERK, p38) and NF-κB (p65) signaling pathways by assessing the phosphorylation status of ERK, p38, and p65 proteins. Western blot analysis revealed that *E. coli* infection enhanced the phosphorylation of these signaling molecules, whereas treatment with MF63 or MK886 significantly suppressed their phosphorylation levels (*p* < 0.01, Figures 2F–2I). These findings suggest that MF63 and MK886 may suppress the secretion of pro-inflammatory cytokines and chemokines while promoting the release of anti-inflammatory mediators during *E. coli* infection, potentially by inhibiting *E. coli*-induced activation of the MAPK and NF-κB signaling pathways in BMDM.

### 3.3 Analysis of the effect of mPGES-1-PGE₂ axis inhibition on the phagocytic and bactericidal activity of BMDM against *Escherichia coli*

**Figure 3:**
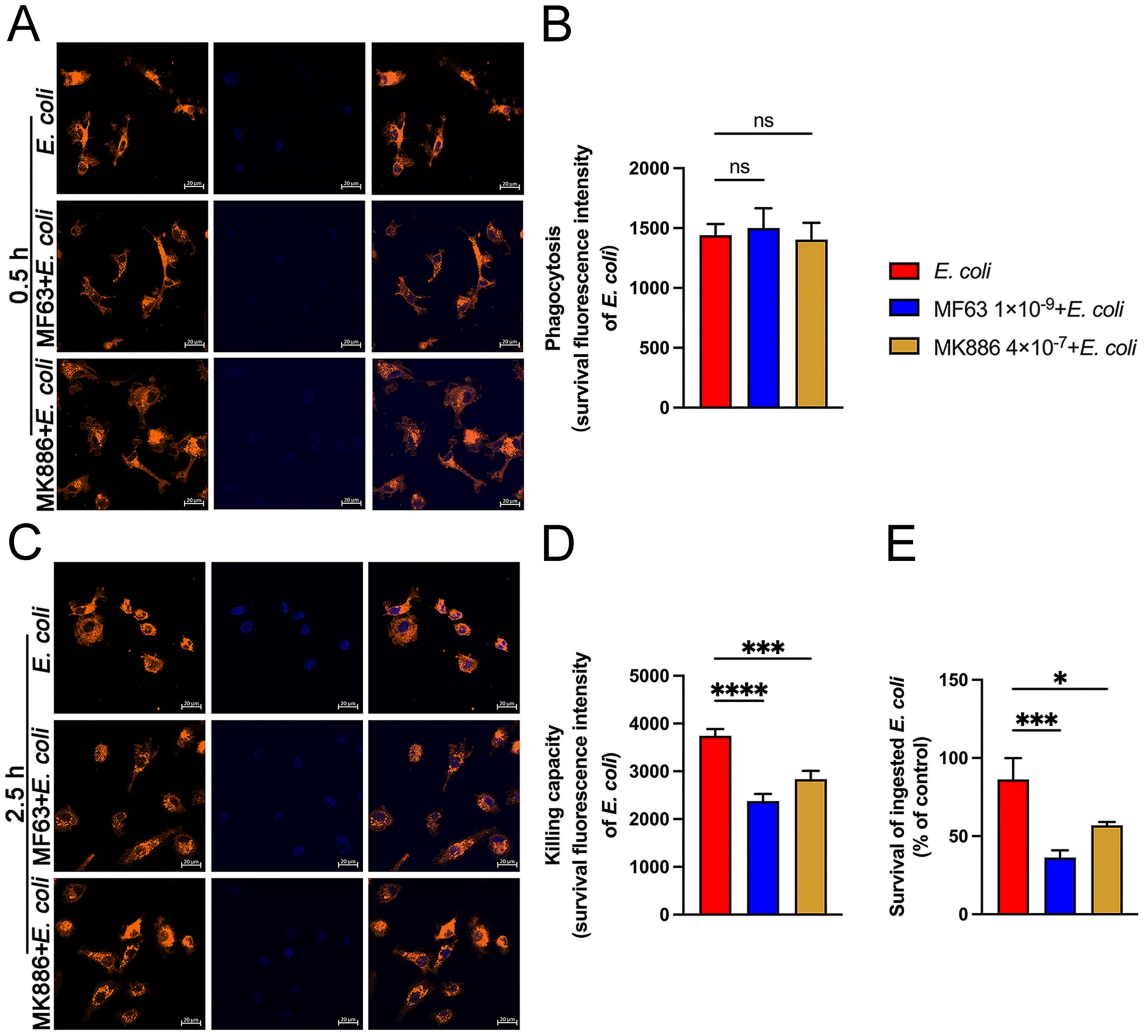
Effects of MF63 and MK886 on the phagocytosis and killing of *E. coli* by BMDMs. **(A,B)** BMDMs were pretreated with MF63 and MK886 for 24 h, followed by infection with *E. coli* (MOI 5:1) for 0.5 h. Phagocytosis of Hoechst 33258-labeled *E. coli* (blue) by DiI-labeled BMDMs (orange) was analyzed using microscopy at ×400 magnification (scale bar = 20 μm). **(C,D)** Effect of MF63 and MK886 on the bactericidal capacity of BMDMs against *E. coli* under an MOI of 5:1 (scale bar = 20 μm). **(E)** MTT assay was used to assess the effect of MF63 and MK886 on *E. coli* viability in BMDMs. Results are presented as mean ± SD from three independent experiments, analyzed by Tukey’s multiple comparisons and two-way ANOVA. Significance is denoted as *p < 0.05, **p < 0.01, ***p < 0.001, ****p < 0.0001 vs *E. coli* group; ^#^*p* < 0.05, ^##^*p* < 0.01 ^###^*p* < 0.001, ^####^*p* < 0.0001 vs Control group.

Next, we used DiI-labeled BMDM and Hoechst-stained *E. coli* to evaluate the phagocytic and intracellular killing abilities of the BMDM. At 0.5 h post-*E. coli* infection, pretreatment with mPGES-1 inhibitors did not affect the macrophages’ ability to phagocytose *E. coli* (Figures 3A,B). However, at 2.5 h post-infection, confocal laser microscopy showed a significantly reduced fluorescence intensity in the mPGES-1 inhibitor groups compared to the infection group (Figures 3C,D, *P* < 0.001). This indicates that inhibiting PGE_2_ synthesis enhances the macrophages’ ability to eradicate *E. coli*. Consistent with these observations, an MTT assay was used to assess the effect of mPGES-1-PGE_2_ axis inhibition on the survival of intracellular *E. coli*. Inhibition of PGE_2_ synthesis resulted in a reduction of *E. coli* survival in BMDM, further demonstrating that inhibition of PGE_2_ synthesis enhances the macrophages’ bactericidal ability (Figure 3E, *P* < 0.05).

### 3.4 Effect of inhibiting the mPGES-1-PGE_2_ axis on the expression of DAMPs during *Escherichia coli* infection

**Figure 4:**
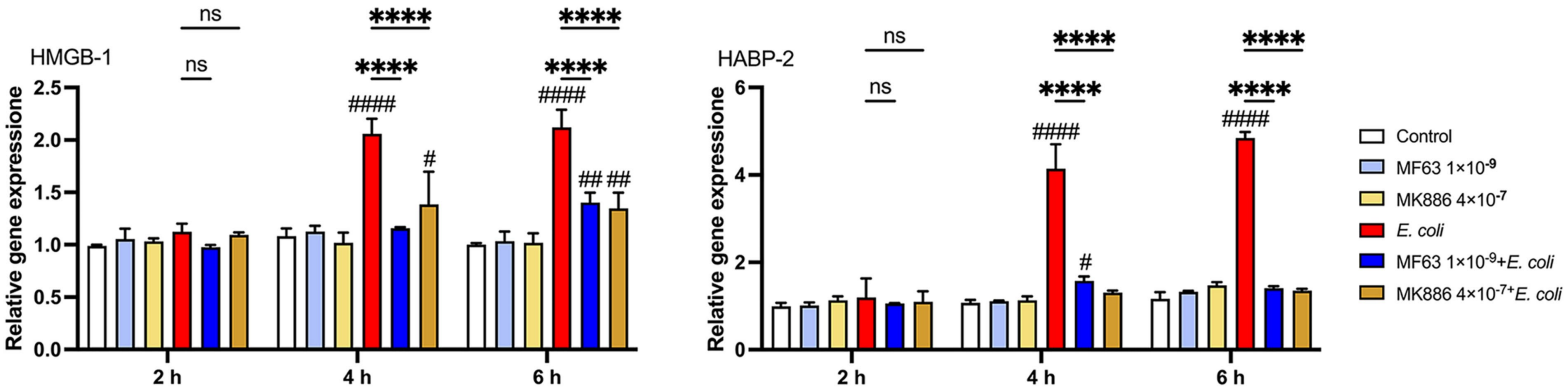
Effect of mPGES-1 inhibitors (MF63 and MK886) on DAMPs in *E. coli*-infected BMDMs. Effect of MF63 and MK886 on *HMGB-1* and *HABP-2* mRNA expression in *E. coli*-infected BMDMs. Results are presented as mean ± SD from three independent experiments, analyzed by Tukey’s multiple comparisons and two-way ANOVA. Significance is denoted as *p < 0.05, **p < 0.01, ***p < 0.001, ****p < 0.0001 vs *E. coli* group; ^#^*p* < 0.05, ^##^*p* < 0.01 ^###^*p* < 0.001, ^####^*p* < 0.0001 vs Control group.

To investigate the effect of inhibiting the mPGES-1–PGE₂ axis on the expression of DAMPs during *E. coli* infection, we measured the mRNA levels of key DAMPs, such as HMGB-1 and HABP-2, in BMDM following treatment with mPGES-1 inhibitors (MF63 and MK886). *E. coli* infection significantly increased the expression of both *HMGB-1* and *HABP-2* in BMDM. However, treatment with mPGES-1 inhibitors notably reduced the mRNA expression levels of these DAMPs at 4 and 6 h post-infection, compared to the *E. coli* infection group (Figure 4, *p* < 0.0001). This suggests that inhibition of the mPGES-1–PGE₂ axis modulates the expression of DAMPs, potentially mitigating inflammation and tissue damage during *E. coli* infection.

### 3.5 Selection of drug concentrations of EP4 inhibitor in *Escherichia coli* infected BMDM

**Figure 5:**
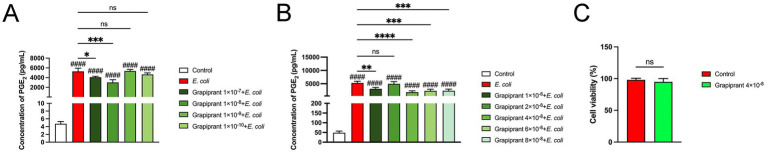
Grapiprant drug selection results. **(A,B)** Effect of varying concentrations of Grapiprant on PGE₂ secretion in *E. coli*-infected BMDMs. **(C)** Cell viability of Grapiprant (4 × 10^−8^ M)-treated BMDMs was assessed using the MTT assay. Results are presented as mean ± SD from three independent experiments, analyzed by Tukey’s multiple comparisons and two-way ANOVA. Significance is denoted as *p < 0.05, **p < 0.01, ***p < 0.001, ****p < 0.0001 vs *E. coli* group; ^#^*p* < 0.05, ^##^*p* < 0.01 ^###^*p* < 0.001, ^####^*p* < 0.0001 vs Control group.

The EP4 inhibitor (Grapiprant), tested at concentrations ranging from 1 × 10^−7^ M to 1 × 10^−10^ M, was applied to *E. coli*-infected BMDM. The concentration of 1 × 10^−8^ M was identified as the most effective in influencing PGE_2_ secretion (Figure 5A, *P* < 0.001). Further refinement revealed the lowest secretion of PGE_2_ at a concentration of 4 × 10^−8^ (Figure 5B, *P* < 0.01). Accordingly, Grapiprant (4 × 10^−8^ M) was chosen as the optimal drug concentration for treating endometritis in dairy cows in subsequent experiments. In addition, MTT results showed that Grapiprant exerted no toxic effects on BMDM (Figure 5C).

### 3.6 Analysis of cytokines and signaling pathways of *Escherichia coli* infected BMDM by inhibiting the PGE_2_-EP4 axis

**Figure 6:**
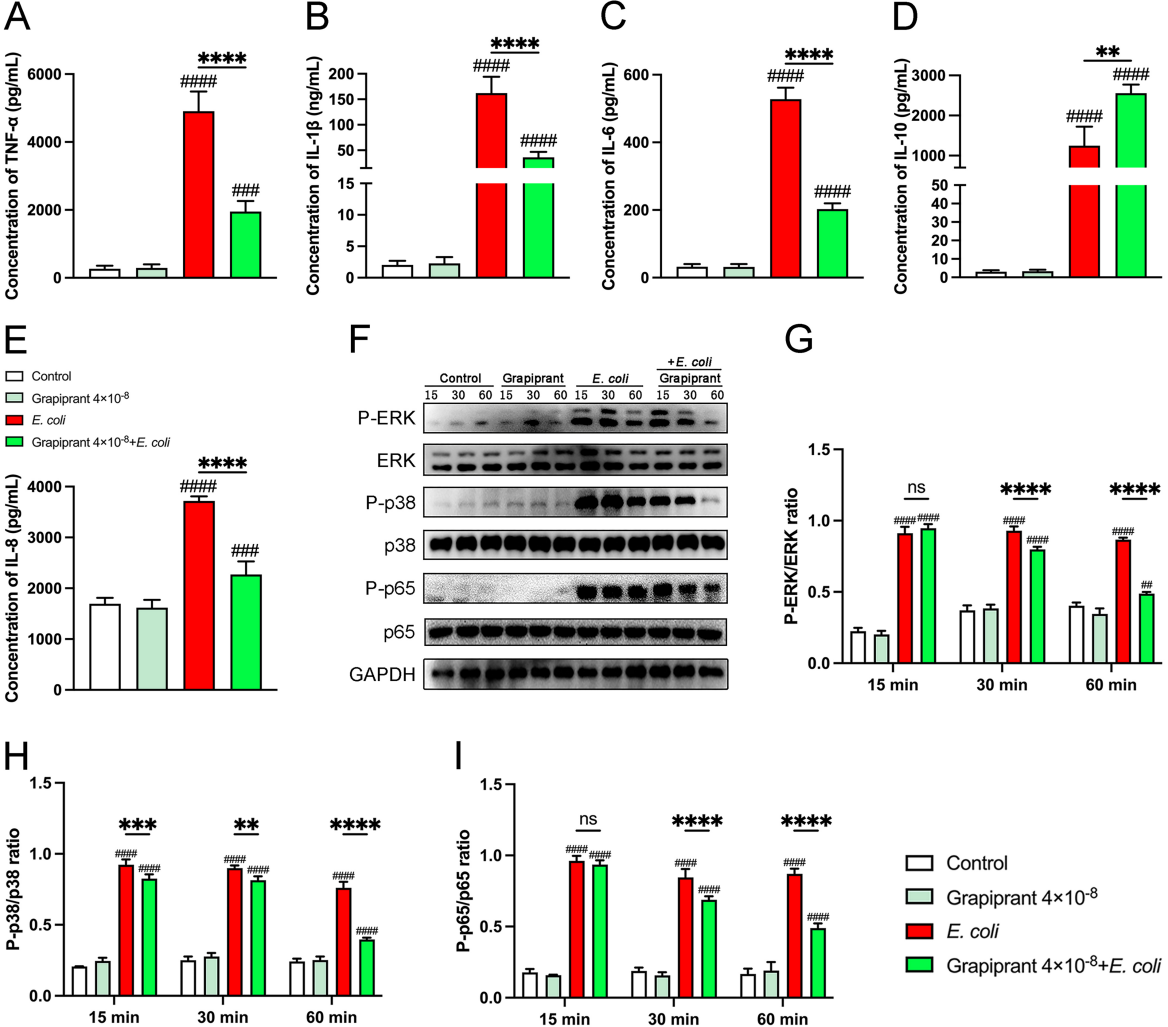
Impact of EP4 inhibitor (Grapiprant) on cytokine secretion and signaling pathways in *E. coli*-infected BMDMs. **(A–E)** BMDMs were pretreated with Grapiprant and subsequently infected with *E. coli* at a MOI of 5:1. The secretion levels of TNF-α, IL-1β, IL-6, IL-10, and IL-8 in the BMDMs culture supernatants were measured by ELISA. **(F–I)** Phosphorylation levels of ERK, p38, and p65 were assessed by western blotting at 15-, 30-, and 60-min post-infection, with GAPDH used as the loading control. Grayscale values were quantified using ImageJ software. Results are presented as mean ± SD from three independent experiments, analyzed by Tukey’s multiple comparisons and two-way ANOVA. Significance is denoted as *p < 0.05, **p < 0.01, ***p < 0.001, ****p < 0.0001 vs *E. coli* group; ^#^*p* < 0.05, ^##^*p* < 0.01 ^###^*p* < 0.001, ^####^*p* < 0.0001 vs Control group.

To investigate the role of the PGE_2_-EP4 pathway in inflammation, we assessed the impact of the EP4 receptor inhibitor, Grapiprant, on cytokine production in *E. coli*-infected BMDM. *E. coli* infection significantly enhanced the secretion of pro-inflammatory cytokines and chemokine in BMDM. However, Grapiprant treatment markedly reduced the secretion of pro-inflammatory cytokines (TNF-*α*, IL-1β, and IL-6) and the chemokine IL-8, while significantly increasing the secretion of the anti-inflammatory cytokine IL-10 in *E. coli*-infected BMDM (Figures 6A–E, *P* < 0.01). Furthermore, we examined the effect of the PGE_2_-EP4 receptor pathway on the activation of MAPK and NF-κB signaling in *E. coli*-infected BMDM, performed through Western blotting, indicated that Grapiprant treatment significantly reduced the phosphorylation of ERK, p38, and p65 compared to the *E. coli* infection group (Figures 6F–I, *P* < 0.01), indicating that the PGE_2_-EP4 pathway modulates these keys signaling pathways in the inflammatory response.

### 3.7 Analysis of the effect of PGE₂-EP4 axis inhibition on the phagocytic and bactericidal activity of BMDM against *Escherichia coli*

**Figure 7:**
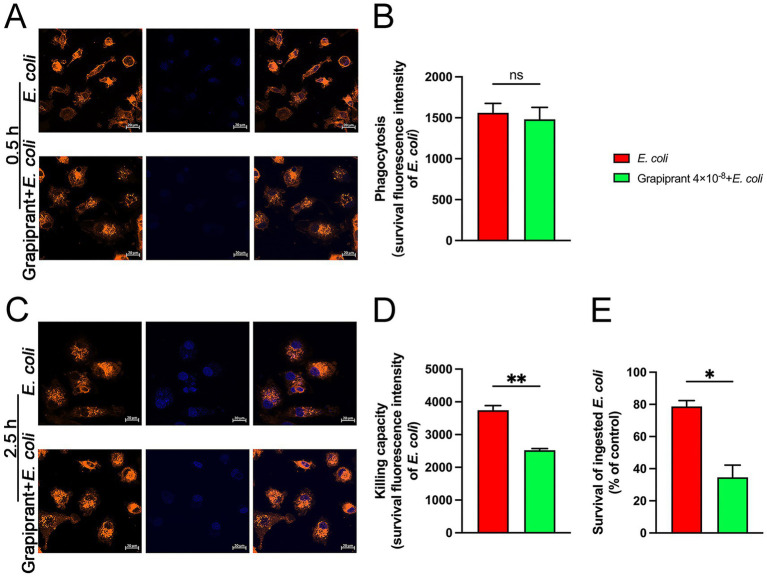
Effects of EP4 inhibitor (Grapiprant) on the phagocytosis and killing of *E. coli* by BMDMs. **(A,B)** BMDMs were pretreated with Grapiprant for 4 h, followed by infection with *E. coli* (MOI 5:1) for 0.5 h. Phagocytosis of Hoechst 33258-labeled *E. coli* (blue) by DiI-labeled BMDMs (orange) was analyzed using microscopy at ×400 magnification (scale bar = 20 μm). **(C,D)** Effect of Grapiprant on the bactericidal capacity of BMDMs against *E. coli* under an MOI of 5:1 (scale bar = 20 μm). **(E)** MTT assay was used to assess the effect of Grapiprant on *E. coli* viability in BMDMs. Results are presented as mean ± SD from three independent experiments, analyzed by Tukey’s multiple comparisons and two-way ANOVA. Significance is denoted as *p < 0.05, **p < 0.01, ***p < 0.001, ****p < 0.0001 vs *E. coli* group; ^#^*p* < 0.05, ^##^*p* < 0.01 ^###^*p* < 0.001, ^####^*p* < 0.0001 vs Control group.

At 0.5 h post-*E. coli* infection, Grapiprant did not significantly affect the phagocytic ability of macrophages compared to the *E. coli* infection group (Figures 7A,B). However, at 2.5 h post-infection, a notable reduction in fluorescence intensity was observed in the Grapiprant treated group (Figures 7C,D, *P* < 0.01). These results suggest that inhibition of the PGE_2_-EP4 receptor pathway enhances the bactericidal activity of macrophages against *E. coli*. Additionally, the MTT assay was employed to evaluate the impact of the PGE_2_-EP4 receptor pathway on the survival of intracellular *E. coli*. In line with the immunofluorescence findings, Grapiprant reduced the survival rate of *E. coli* in BMDM, further confirming that targeting the PGE_2_-EP4 receptor pathway enhances the bactericidal capacity of BMDM (Figure 7E, *P* < 0.05).

### 3.8 Analysis of the effect of PGE₂-EP4 axis inhibition on the phagocytic and bactericidal activity of BMDM against *Escherichia coli*

**Figure 8:**
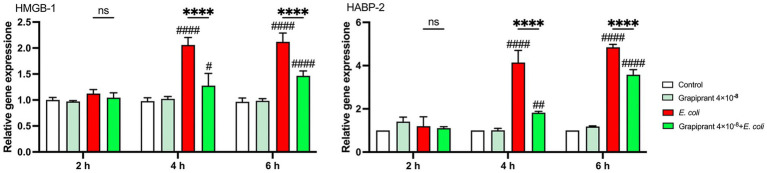
Effect of EP4 inhibitor (Grapiprant) on DAMPs in *E. coli*-infected BMDMs. Effect of Grapiprant on *HMGB-1* and *HABP-2* mRNA expression in *E. coli*-infected BMDMs. Results are presented as mean ± SD from three independent experiments, analyzed by Tukey’s multiple comparisons and two-way ANOVA. Significance is denoted as *p < 0.05, **p < 0.01, ***p < 0.001, ****p < 0.0001 vs *E. coli* group; ^#^*p* < 0.05, ^##^*p* < 0.01 ^###^*p* < 0.001, ^####^*p* < 0.0001 vs Control group.

To examine the impact of inhibiting the PGE_2_-EP4 axis on DAMPs expression during *E. coli* infection, we assessed the mRNA expression of HMGB-1 and HABP-2 in BMDM. The results showed that *E. coli* infection significantly upregulated the expression of both *HMGB-1* and *HABP-2* compared to the control group. However, treatment with Grapiprant led to a notable reduction in the mRNA expression levels of HMGB-1 and HABP-2 at 4 and 6 h post-infection (Figure 8, *p* < 0.0001). These findings indicate that inhibition of the PGE_2_-EP4 pathway can modulate the release of DAMPs during the inflammatory response to *E. coli* infection.

## 4 Discussion

This study was conducted to elucidate the effects of mPGES-1 inhibitors and EP4 receptor antagonists on inflammatory responses mediated by *E. coli* infection in BMDM. The key findings revealed that these targeted interventions significantly reduced PGE₂ levels, mitigated the activation of inflammatory signaling pathways, and enhanced macrophage bactericidal activity, thereby demonstrating potential as viable alternatives to conventional antibiotic therapy in managing mastitis.

Milk, one of the most essential animal-derived food products, plays a crucial role in meeting the nutritional needs of humans (1). The performance of dairy cows, particularly regarding milk production and quality, directly impacts dairy supply and the sustainability of the food industry (37). However, mastitis, a common postpartum infection of the mammary gland, severely affects mammary health, leading to decreased milk production and quality, and causing significant economic losses in dairy farming (38). Mastitis development is influenced by various factors, with pathogenic bacterial infections being a primary contributor. Among these, *E. coli* is a major pathogen responsible for mastitis (7). Through the release of virulence factors, such as lipopolysaccharides, adhesins, and toxins, *E. coli* induces damage and inflammatory responses in mammary tissues, potentially leading to abscess formation and further loss of mammary function in severe cases (39). However, the pathogenesis of *E. coli*-induced mastitis remains inadequately understood. This study demonstrated that *E. coli* infection significantly elevated PGE₂ secretion in macrophages. As a key inflammatory mediator, PGE₂ plays a critical role in the progression of mastitis (18). Currently, antibiotic treatment is still the main means of controlling mastitis in dairy cows, but the health risks associated with its long-term use cannot be ignored. In addition, antibiotic use may adversely affect the intestinal flora of dairy cows, which in turn affects the immune system’s response and exacerbates the onset and progression of mastitis (40, 41). Consequently, exploring alternative therapeutic strategies targeting PGE₂ may provide a novel approach to treat mastitis, reduce reliance on antibiotics, and minimize their adverse effects on both health and immune function in humans.

PGE₂ is widely present in various tissues and organs in both humans and animals, where its synthesis and release typically respond to stimuli such as infection and inflammation (42). PGE₂, a biologically active lipid, is synthesized from AA by COX and different isoforms of PGES, with mPGES-1 being the most critical. mPGES-1 is co-expressed with COX-2 and plays a significant role in inflammatory processes by catalyzing the conversion of PGH₂ to PGE₂, thereby enhancing the inflammatory response (43). Given its crucial role, mPGES-1 inhibition has been explored as a strategy to modulate PGE₂ levels and attenuate inflammation. For instance, inhibitors like MF63 and MK886 reduce PGE₂ synthesis, alleviating the excessive inflammatory response (44, 45). Consistent with the findings of this study, both MF63 and MK886 significantly reduced PGE₂ secretion in *E. coli*-infected BMDM, indicating their effective inhibition of PGE₂'s pro-inflammatory role. However, the precise mechanisms by which these inhibitors affect the onset and progression of mastitis remain unclear. Further research is necessary elucidate the therapeutic potential of mPGES-1 inhibitors in mastitis and provide a foundation for developing novel anti-inflammatory therapies.

In conditions such as mastitis, the release of pro-inflammatory cytokines, including TNF-*α*, IL-1β, IL-6, and IL-8, significantly intensifies the inflammatory response, thereby exacerbating tissue damage and pathological alterations (46, 47). Additionally, when cells are exposed to pathogenic factors, the MAPK signaling pathways (e.g., ERK, p38) are activated. This activation triggers a cascade of kinases, ultimately leading to the activation of transcription factors such as NF-κB in the cell nucleus, which in turn regulates the transcription of pro-inflammatory cytokines and chemokines (48). *In vitro* studies have shown that sulforaphane downregulates the mRNA expression of inflammatory cytokines, inhibits the expression of inflammatory mediators such as COX-2 and inducible nitric oxide synthase, and suppresses NF-κB activation, thereby alleviating LPS-induced mastitis (49). Consistent with these findings, our study demonstrated that MF63 and MK886 significantly reduced the secretion of TNF-α, IL-1β, IL-6 and IL-8 in *E. coli*-infected BMDM, while significantly increasing the secretion of the anti-inflammatory cytokine IL-10. Moreover, these compounds effectively inhibited the activation of both NF-κB and MAPK signaling pathways. These results suggest that MF63 and MK886 mitigate inflammation by suppressing PGE_2_ synthesis, thereby inhibiting the activation of NF-κB and MAPK signaling pathways, reducing pro-inflammatory cytokine secretion, and effectively alleviating the inflammatory response. This indicates that MF63 and MK886 play a critical role in mitigating inflammation.

Macrophages are instrumental in immune responses, including pathogen and tumor cell clearance, cytokine production, and intercellular interactions (50). Phagocytosis and intracellular killing are essential steps in bacterial clearance. Phagocytosis involves the internalization of bacteria, while subsequent killing mechanisms, activated once bacteria are engulfed, work together to eliminate the pathogens (51). Previous studies have shown that PGE₂ inhibits the bactericidal activity of alveolar macrophages against *Klebsiella pneumoniae* (52) and suppresses H₂O₂ generation during the clearance of apoptotic cells, impairing *Streptococcus pneumoniae* clearance (53). In line with our findings, inhibition of PGE₂ synthesis by MF63 and MK886 did not affect the phagocytic activity of BMDM but significantly enhanced their bactericidal ability against *E. coli*. This suggests that PGE₂ modulates the immune response intensity by affecting macrophage bactericidal function without directly influencing phagocytosis. Similarly, decursinol enhances the bactericidal activity of macrophages without significantly affecting their phagocytic function, as shown in studies against methicillin-resistant *Staphylococcus aureus*, while also reducing excessive pro-inflammatory cytokine expression and the inflammatory response (54). These findings underscore the crucial role of PGE₂ inhibition in regulating inflammation and modulating the effectiveness of the immune response by influencing bacterial clearance.

Following *Mycobacterium tuberculosis* infection of M1 macrophages, lactate significantly reduced bacterial load and alleviated tissue damage by enhancing macrophage clearance ability (55). Similarly, polysaccharides from *Codonopsis pilosula* (a perennial flowering plant in the bellflower family) improve macrophage pathogen clearance, thereby reducing bacterial load and mitigating pulmonary pathology (56). These findings highlight the close relationship between macrophage killing ability and tissue damage. During inflammation, the massive release of pro-inflammatory cytokines and chemokines promotes immune cell recruitment and activation, while inducing oxidative stress, extracellular matrix degradation, and increased vascular permeability, all of which exacerbate tissue damage. High mobility group box 1 (HMGB-1) and hyaluronan-binding protein-2 (HABP-2)—two key damage-associated molecular patterns (DAMPs)—are released during cell damage or death, triggering inflammation. HMGB-1 is released from the nucleus into the extracellular space, while HABP-2 is upregulated following tissue injury, promoting tissue repair through cell adhesion and immune cell function (57, 58). In this study, the mRNA expression of HMGB-1 and HABP-2 in *E. coli*-infected BMDM was significantly reduced, suggesting that inhibiting PGE_2_ synthesis may alleviate tissue damage and improve mammary health by modulating DAMP release. Consistent with the results of this study, *in vitro* study has shown that mPGES-1 exacerbates neuronal injury by producing PGE_2_ (59). Furthermore, MF63 and MK886 alleviated endometrial damage in *E. coli*-infected bovine tissue by inhibiting PGE_2_ synthesis and blocking DAMP expression (60).

PGE₂ is an important bioactive molecule that exerts its effects through binding to four G-protein-coupled receptors (EP1, EP2, EP3, and EP4), with the EP4 receptor playing a crucial role in immune responses and inflammation (61). Grapiprant, a selective EP4 receptor antagonist, is widely used in treating PGE₂-mediated inflammatory conditions, particularly in arthritis management in dogs. In animal models, Grapiprant has shown promise in alleviating pain and improving function (62). However, its role and mechanism in *E. coli*-induced mastitis remain poorly understood. Peptidoglycan induces cytokine production in RAW 264.7 macrophages via the PGE₂-EP4-NF-κB and PGE₂-EP4-MAPK pathways (63), and PGE₂ inhibits lipopolysaccharide-induced cytokine production in macrophages by suppressing EP4 signaling, likely through blocking NF-κB activation (64). In this study, Grapiprant inhibited PGE₂-EP4 signaling, leading to reduced activation of NF-κB and MAPK pathways in *E. coli*-induced BMDM. This resulted in decreased secretion of pro-inflammatory cytokines and chemokines, while significantly increasing the secretion of the anti-inflammatory cytokine IL-10. Additionally, Grapiprant enhanced the bactericidal activity of BMDM against *E. coli* and reduced the expression of DAMPs. These findings highlight the significant anti-inflammatory potential of targeting PGE₂-EP4 signaling in the inhibition of *E. coli*-induced mastitis. A previous study has shown that PGE₂ induces T helper 1 (Th1) cell differentiation and Th17 cell expansion *in vitro*. Treatment with an EP4 inhibitor reduces Th1 and Th17 cell accumulation in regional lymph nodes, thereby inhibiting the progression of chronic inflammation (65). Additionally, the EP4 inhibitor suppresses pro-inflammatory cytokine IL-6, chemokine CXCL8, and inflammation-dependent bone metastasis, while alleviating immune suppression and restoring anti-tumor immunity (66). Furthermore, the EP4 antagonist significantly reduces peritoneal fibrosis and improves dysfunction by inhibiting NLRP3 inflammasome and p-p65-mediated inflammatory responses (67). These findings, consistent with our own, underscore the critical role of the PGE₂-EP4 signaling pathway in inflammation and its potential as a therapeutic target.

This study has some limitations. The experimental design primarily focused on in vitro studies, and therefore, further validation through *in vivo* experiments is necessary.

## 5 Conclusion

This study systematically investigated the role of mPGES-PGE₂-EP4 pathway in mastitis pathogenesis during *E. coli* infection and evaluated the therapeutic value of mPGES-1 inhibitors (MF63, MK886) and EP4 receptor antagonists (Grapiprant). *E. coli* infection significantly induced PGE₂ synthesis and release in BMDM, activating NF-κB and MAPK pathways, upregulating pro-inflammatory cytokines and chemokines, thereby exacerbating inflammation and tissue damage. Moreover, PGE₂ impairs macrophage pathogen-killing ability, reducing the host’s efficiency in pathogen clearance. Application of MF63, MK886, and Grapiprant significantly reduces PGE₂ levels, inhibits NF-κB and MAPK activation, decreases inflammatory factor secretion, and enhances macrophage bactericidal capacity, thereby demonstrating anti-inflammatory and immunoregulatory effects. Additionally, inhibiting mPGES-PGE₂-EP4 signaling pathway reduces DAMP expression, such as HMGB-1 and HABP-2, suggesting its role in alleviating *E. coli*-induced damage and improving udder health. These findings not only deepen our understanding of the molecular mechanisms underlying *E. coli*-induced mastitis but also highlight the mPGES-PGE₂-EP4 pathway as a promising therapeutic target. This work provides new insights into the development of targeted anti-inflammatory strategies, offering potential benefits for improving dairy cow health, reducing antibiotic reliance, and promoting the sustainable development of the dairy industry.

## Data Availability

The datasets presented in this study can be found in online repositories. The names of the repository/repositories and accession number(s) can be found in the article/supplementary material.
